# Supplementary material for: The development and diversity of religious cognition and behavior: Protocol for Wave 1 data collection with children and parents by the Developing Belief Network
Source: PLoS One. 2024 Mar 8;19(3):e0292755. doi: 10.1371/journal.pone.0292755 (PMC10923471; doi:10.1371/journal.pone.0292755)
Supplement: S1 Text — (DOCX) [file pone.0292755.s001.docx]

# **Supporting Information**

For the full text of all materials, all visual stimuli, and Qualtrics surveys, see the following OSF repository: <https://osf.io/dumf4/>.
