# Supplementary material for: The development and diversity of religious cognition and behavior: Protocol for Wave 1 data collection with children and parents by the Developing Belief Network
Source: PLoS One. 2024 Mar 8;19(3):e0292755. doi: 10.1371/journal.pone.0292755 (PMC10923471; doi:10.1371/journal.pone.0292755)
Supplement: Attachment — Submitted filename: Response to Reviewers.pdf [file pone.0292755.s002.pdf]

Response to Reviewers

*Please note that our responses to reviewers are provided in **blue**, and all page numbers refer to the "tracked changes" version of the revised manuscript.*

Journal requirements:

1. Please ensure that your manuscript meets PLOS ONE's style requirements, including those for file naming. The PLOS ONE style templates can be found at https://journals.plos.org/plosone/s/file?id=wjVg/PLOSOne_formatting_sample_main_body.pdf and https://journals.plos.org/plosone/s/file?id=ba62/PLOSOne_formatting_sample_title_authors_affiliations.pdf

We have edited the manuscript to meet the journal's style requirements.

2. Please note that in order to use the direct billing option the corresponding author must be affiliated with the chosen institute. Please either amend your manuscript to change the affiliation or corresponding author, or email us at plosone@plos.org with a request to remove this option.

We have changed the corresponding author to Kara Weisman, who is affiliated with the University of California, Riverside.

3. "One of the noted authors is a group or consortium [insert name of group or team]. In addition to naming the author group, please list the individual authors and affiliations within this group in the acknowledgments section of your manuscript. Please also indicate clearly a lead author for this group along with a contact email address.

We now list the individual authors and affiliations and indicate the two leaders of this group (with contact email addresses) in the Acknowledgements section of the manuscript.

4. Your ethics statement should only appear in the Methods section of your manuscript. If your ethics statement is written in any section besides the Methods, please move it to the Methods section and delete it from any other section. Please ensure that your ethics statement is included in your manuscript, as the ethics statement entered into the online submission form will not be published alongside your manuscript.

We have double checked that ethics-related statements only appear in the Methods section of the manuscript.

5. We note that [Figure 1] in your submission contain [map/satellite] images which may be copyrighted. All PLOS content is published under the Creative Commons Attribution License (CC BY 4.0), which means that the manuscript, images, and Supporting Information files will be freely available online, and any third party is permitted to access, download, copy, distribute, and use these materials in any way, even commercially, with proper attribution. For these reasons, we cannot publish previously copyrighted maps or satellite images created using proprietary data, such as Google software (Google Maps, Street View, and Earth). For more information, see our copyright guidelines:
http://journals.plos.org/plosone/s/licenses-and-copyright.

We require you to either (1) present written permission from the copyright holder to publish these figures specifically under the CC BY 4.0 license, or (2) remove the figures from your submission:

a. You may seek permission from the original copyright holder of Figure(s) [#] to publish the content specifically under the CC BY 4.0 license. We recommend that you contact the original copyright holder with the Content Permission Form (http://journals.plos.org/plosone/s/file?id=7c09/content-permission-form.pdf) and the following text: “I request permission for the open-access journal PLOS ONE to publish XXX under the Creative Commons Attribution License (CCAL) CC BY 4.0 (http://creativecommons.org/licenses/by/4.0/). Please be aware that this license allows unrestricted use and distribution, even commercially, by third parties. Please reply and provide explicit written permission to publish XXX under a CC BY license and complete the attached form.” Please upload the completed Content Permission Form or other proof of granted permissions as an ""Other"" file with your submission. In the figure caption of the copyrighted figure, please include the following text: “Reprinted from [ref] under a CC BY license, with permission from [name of publisher], original copyright [original copyright year].

b. If you are unable to obtain permission from the original copyright holder to publish these figures under the CC BY 4.0 license or if the copyright holder’s requirements are incompatible with the CC BY 4.0 license, please either i) remove the figure or ii) supply a replacement figure that complies with the CC BY 4.0 license. Please check copyright information on all replacement figures and update the figure caption with source information. If applicable, please specify in the figure caption text when a figure is similar but not identical to the original image and is therefore for illustrative purposes only. The following resources for replacing copyrighted map figures may be helpful: USGS National Map Viewer (public domain): http://viewer.nationalmap.gov/viewer/ The Gateway to Astronaut Photography of Earth (public domain): http://eol.jsc.nasa.gov/sseop/clickmap/ Maps at the CIA (public domain): https://www.cia.gov/library/publications/the-world-factbook/index.html and https://www.cia.gov/library/publications/cia-maps-publications/index.html NASA Earth Observatory (public domain): http://earthobservatory.nasa.gov/ Landsat: http://landsat.visibleearth.nasa.gov/ USGS EROS (Earth Resources Observatory and Science (EROS) Center) (public domain): http://eros.usgs.gov/# Natural Earth (public domain): http://www.naturalearthdata.com/.

The map featured in Figure 1 uses open source data from OpenStreetMap® licensed under the Open Data Commons Open Database License (ODbL) by the OpenStreetMap Foundation (OSMF).

Per the OpenStreetMap copyright and license guidelines (link: https://www.openstreetmap.org/copyright), "You are free to copy, distribute, transmit and adapt our data, as long as you credit OpenStreetMap and its contributors. If you alter or build upon our data, you may distribute the result only under the same license. [...] Our documentation is licensed under the Creative Commons Attribution-ShareAlike 2.0 license (CC BY-SA 2.0)." We have included additional language about this attribution in the caption for Figure 1.

Please review your reference list to ensure that it is complete and correct. If you have cited papers that have been retracted, please include the rationale for doing so in the manuscript text, or remove these references and replace them with relevant current references. Any changes to the reference list should be mentioned in the rebuttal letter that accompanies your revised manuscript. If you need to cite a retracted article, indicate the article's retracted status in the References list and also include a citation and full reference for the retraction notice.

(Please note that all page numbers refer to the "tracked changes" version of the revised manuscript.)

We have added the following new references: Barrett (2012) (cited on page 4); Schwitzgebel (2021) (page 5); Rogoff, Dahl, & Callanan (2018) (page 21); Hay & Nye (2006) (page 31); Heiphetz, Lane, Waytz, & Young (2016) (page 31); Diesendruck & HaLevi (2006) (page 37); Diesendruck (2020) (page 37); Taylor et al. (2021) (page 37); Heiphetz, Spelke, & Banaji (2013) (pages 51, 54); Takriti (2002) (pages 51, 54); Elkind (1964) (pages 51, 54); Quintana (1998) (page 54); Payir et al. (2021) (page 57); Vaden & Woolley (2011) (page 57).

We do not cite any retracted papers.

Reviewer Comments

Reviewer #1:

The authors may want to consider expanding the data collection regions to account for differences in micro-cultures. For instance, in the US, data are being collected in California and Massachusetts, two of the least religious and most socio-politically liberal regions in the US, but not in the US South, which is the most religious region and the most socio-politically conservative. I assume this would extend to other collection regions, as well.

The decision to target samples from Southern California and Massachusetts is driven primarily by feasibility for longitudinal data collection (with the potential for in-person work in future waves of data collection). Within each of these geographical regions, our planned samples are not simply convenience samples; the data collection teams are going to great lengths to recruit religious participants from multiple religious traditions (as well as non-religious participants).

More broadly, however, we agree with the reviewer. In the future, we hope to secure additional resources that would allow us to expand to include more geographical regions both within the US and within other countries represented in the network.

Reviewer #2:

The researchers have put forth an amendable effort to create, elaborate and document an extensive protocol addressing previously under-explored segments of developing child religiosity This vast and notable effort has well-elaborated goals presented with extensive references that support their conclusions and point to the gap in the knowledge.

It also demonstrates the meticulous planning and preparation that has gone into this research project which resulted in a well-structured report.

We thank Reviewer #2 for their interest in and appreciation of this large scale research endeavor.

There are only a handful of minor items and structural issues that I would encourage the researchers to consider:

1. Citing dr. Barret in paragraph two on the religious cognitive development would seem appropriate (i.e., Barrett, J. L. (2012). Born believers: The science of children's religious belief. Simon and Schuster.)

Thank you for suggesting this. We have added this citation to paragraph 2 on page 4.

(Please note that all page numbers refer to the “tracked changes” version of the revised manuscript.)

2. Bracket missing in front of reference nr. 9 in the text

Thank you for catching this. We have corrected the formatting of this in-text citation on page 4.

3. The segment Roadmap to current manuscript, although informative, is colloquial in form and lacks the tone that has been set beforehand and should be edited to better reflect the style of a scientific paper.

We have revised this section to be more formal in tone and changed the subheading to “Manuscript overview” (page 6).

4. Subgoals for each of the project aims would do well to be equally bulleted for clarity.

Thank you for this suggestion. We ultimately decided to retain the organization and format as we had it, in part because the subgoals are numbered within the text. Therefore, we believe that the reader will be able to understand our process. Nevertheless, we are happy to provide bullets if the Editor deems it appropriate.

5. In the Materials and Methods segment the authors state that: „Sample-specific item-selection, sample size, eligibility, inclusion and exclusion criteria, recruitment strategies, the mode of data collection, and compensation vary across samples and will be detailed in future manuscripts.”
It would be advisable to inform the reader more clearly at this point that these segments are available in this report for the US sample and that they will be available in further manuscripts for other samples. If there are some guidelines that direct an overarching strategy for determining these criteria and strategies, they could also be laid out in this segment.

Thank you for this suggestion. We have added some additional clarification that this manuscript handles adaptation and implementation in the US on page 15.

6. In the same segment where exclusion criteria for the US sample are elaborated, it is stated that parental assessments of working memory can lead to exclusion. Does this translate to measured working memory limitations also? If yes, the criteria should be included. In both cases, exclusion criteria should be more transparent to allow for successful replication of the

protocol.

Thank you for this suggestion. The exclusion criteria for the US sample does not include exclusion based on formal measurements of working memory (through the Memory Span tasks) or other formal assessments of cognitive limitations. This exclusion criteria pertains only to children whose parents spontaneously self-report concerns about their child's comprehension, verbal expression, working memory, or other factors related to their child's ability to complete the study. We have added some wording to make the clarification more explicit (see page 18).

7. The wording of the Exclusion criteria paragraph stating that it is representative of the US sample is somewhat confusing and should in style reflect the paragraph on Inclusion criteria in the Sample segment of the paper.

We are unsure which paragraph the reviewer is referring to, but have made several revisions throughout pages 15-18 that we hope have improved the clarity of our inclusion and exclusion criteria.

8. Please elaborate on the rationale behind using optional caregiver tasks.

Of course. The tasks that are currently marked as Optional were originally part of the standard caregiver protocol (i.e., they were required). However, this resulted in a caregiver protocol that was too long for some field sites/samples. To strike a balance between research interests and feasibility, we decided to make a distinction between required and optional tasks for caregivers, and allow research teams to opt into (or out of) including optional tasks. We now provide further clarification in the manuscript (page 68).

9. It would be advisable to also report the expected/experienced time to collect the answers within each protocol and for each age bracket.

Thank you for this suggestion. We have added this information about estimated task duration for the youngest participants and oldest participants at the end of each task description (pages 25, 30, 35, 40, 46, 53, 56, 60, 66, 79).

10. The report on page 22 states that research teams were encouraged to use a mix "of specific individuals (i.e., Santa Claus) and plural generics (i.e., Dragons) as well as sample specific items in the list". Research question 2, the Religious Indicators task uses similar language where it states task properties vary and "might" focus on different qualities. For instance, the first paragraph on page 31 states: "The properties included in this task might focus on the character individually (e.g., "Here is a kid who...") or on the character's family (e.g., "Here is a kid whose family...") as deemed appropriate in a given setting. Research teams have been encouraged,

but not required, to select religious properties that differentiate among salient religious groups in the local context. (e.g., choosing a belief about Jesus rather than God in settings where Christians live alongside members of other monotheistic religions); the only strict requirement is that these properties should be salient aspects of religious identity and should be familiar and relevant to children, as confirmed by local informants (e.g., religious leaders, parents of young children).”

There are a few more such references that indicate an arbitrary selection of items and stimuli. If that is the case, a more extensive elaboration of the level to which such indiscriminate change to the protocol can be made, as well as a rationale on when and to what extent is the protocol malleable would be beneficial to researchers attempting to replicate these findings and use these protocols.

Thank you for bringing this up. The wording in the original submission may have given the impression that these sample specific adaptations were arbitrary. We have updated the wording to explain that variations in a given task’s item selection across samples were permitted insofar as they improved an item’s cultural relevance and ecological validity. For example, the sorting task has inherent variability because research teams were encouraged to identify agents/entities that are prominent within their samples that were good exemplars of our pre-set definitions (e.g., using the term “God” to represent the most prominent god-like figure for Catholics in the US, “Buddha” for Buddhists in Singapore, “Allah” for Muslims in Lebanon, etc.). See pages 23, 33, 45.

11. The planned analysis segments although informative lack a unifying structure and comparable style. For instance, planned analyses of the sorting task segment (page 24), in the second paragraph, starts with the sentence “We anticipate researchers analysing the data with multiple aims.“ as if to indicate what some other researchers outside of the consortium might be interested in analysing. This, however, is not what is present in other segments where the authors indicate the analysis they are or will be interested in conducting. The planned analysis segment should follow up with the elaboration of the planned analysis that are conducive to answering the research questions. Anticipation of analysis of other researchers should be left out as speculation or could be included as suggestions or recommendations. The Planned Analysis segment of the Property Attributions task (page 28) again faces the same impersonal language and inconsistency. The wording used now is “describing the most basic tasks” but it is not clear what this means. Consider using uniform structure and wording to explain the likelihood and timeline of these analyses.

We have removed the statement “We anticipate researchers analyzing the data with multiple aims” from the planned analysis section for the Sorting task (page 26), and have removed statements about the “most basic analyses” from all planned analysis sections. We have instead added a paragraph in the Introduction orienting the reader to these “Planned analyses” sections (page 6).

We have also edited all of the “Planned analyses” sections to implement a clearer parallel structure across tasks.

12. The tone of the text switches from personal to impersonal. Especially in the planned analysis segment. For instance, in the Moral norm variation analyses the authors state they “might also examine the main effect”. In this form, this information is either superfluous or not appropriately presented. I urge the authors to avoid using “we” and shift to an impersonal style of writing as well as avoid speculating on what someone might or might not do and present what is feasible, advisable, required, or encouraged.

We have revised most sentences in which we formerly used “might” (e.g., pages 34, 35, 39). In places where we formerly speculated about what analyses might be performed, we have either revised the sentence to state what analyses will be performed (e.g., page 50) or omitted the speculation (e.g., pages 42, 58).

After careful consideration, we have maintained the use of the first person plural “we” throughout the manuscript, because we find it to improve the clarity of our descriptions of this complex protocol and the complex processes that contributed to its development.

13. The authors in multiple segments address the need for Coding schemes (i.e., Justification task, Caregiver-Child Conversation task) but do not elaborate sufficiently on this issue for it to be informative for further research. I would propose a more detailed elaboration of the mentioned coding schemes to the scope that is appropriate for a protocol report.

These coding schemes have not yet been developed; where possible, we include references to relevant coding schemes that have been used in previous research, but their application to the current study protocol will require extensive refinement and elaboration to suit our research questions and our diverse range of samples. Indeed, we have been careful to not apply our Western-centric bias to any coding scheme and therefore are recommending holding off on a coding scheme until all data from the first wave has been collected and all researchers are able to verify that it is valid in their setting.

14. In the Planned analyses of Religion questions segment the authors state the analysis “will be qualitative and exploratory in nature; we will not describe them here”. I would advise for the last sentence to be rephrased in order to better reflect a more formal writing style.

We have revised this sentence (page 53).

15. Including references that justify hypotheses put forth in the analysis section would also be advisable.

We have revised all of our "Planned analyses" sections to incorporate additional references to previous work that has tested similar hypotheses, where applicable (pages 26-27, 31, 41-42, 50, 54, 57, 62, 67).

A few additional questions for the researchers:

• Protocols include questions about the COVID pandemic. Is that related to the work and goals, and how? If yes, should it be used by other researchers, and to what end?

We welcome the invitation to clarify why we have included questions about the COVID-19 pandemic and now address this in our description of the Caregiver-Child Conversation task (page 63).

• Should Caregiver-Child Conversation data that was collected independently be coded as such?

We have added a brief note indicating that analyses of data from this task should take into account differences across participants in the administration of the task, i.e., the extent to which it was administered independently (page 66-67).

Finally, I would like to commend the authors for their work. Apart from these minor, and mostly style-based comments, I do not have any substantial issues with the presented work, and I wholeheartedly recommend the publication of this report.

We sincerely thank the reviewer for their helpful suggestions and their thorough engagement with this report.

Reviewer #3:

- The manuscript describes a protocol for epic data collection for the developmental and cross-cultural study of religious thinking and behavior of children (age between 4-10) and their parents across the chosen religious traditions of present times.
- The manuscript seems to be well thought out and contains a clear and concise description of what the authors aim to do, partly why they want to do it and why in that specific way.
- As it is not an article publishing research results and conclusions, where I can consider the relationship between questions and answers, I feel uneasy about how I should actually review the presented work.
- I am used to being in the position of reviewing protocol (study designs) in the situation of considering funding, or where the feedback to authors should help to improve the design. Neither of these criteria seems to be applicable in this case. As it is certainly applaudable to

make the design of the research public as a form of "pre-registration", I am not sure what is the role of peer review for such a thing.
- I generally enjoyed reading the protocol and the opportunity to think about the research problem and the complexities connected with the hardship of studying it. Even though, to my taste, the authors rely too much on a specific understanding of religion (focus on belief) and too much on cognitivist cognitive science in their theoretical and methodological perspective (behaviour is controlled by disembodied mental representations and mechanisms, which can be studied just in conversation and in discursive exercises).
- I believe, that the research can and will bring exciting new findings, I identify with the authors' claim that children's religious worldviews are an understudied phenomenon. But I do not identify with some theoretical and methodological choices. I am, however, reluctant to classify such differences as the author's problems or mistakes, which should anyhow hinder the publication or their research.

We thank the reviewer for engaging with this research so thoroughly, and we are glad to see that the reviewer agrees that this research will bring exciting new findings.

- Further notes are then brief, they point more to my experience with the text than the text itself:
- The first task for children puzzles me, and I admit there lies a root of my personal uncomfort about the design of the study. The "what is real" problem as part of the autonomous NON-4E (enacted, embodied, embedded, extended) cognition belief system is something which I connect to the beginnings of the cognitive science of religion, and I am surprised to see it in such a naked form after 20 years of existence of its empirical research program and at least a decade of various critique why especially in the case of religion, we should NOT use the methods of the laboratory cognitive psychology from the eighties.

We agree that most of the tasks in the current protocol use methods that are characteristic of the cognitivist orientation of early CSR research, and we acknowledge that these methods do not provide a complete picture of conceptual development in this domain. However, these methods remain common in the field of developmental psychology, and reveal information about how children ontologically organize these kinds of concepts. We feel that an extensive discussion of the theoretical underpinnings of this work is outside the scope of the current, methodological manuscript, especially given its current length. However, we have added a brief overview of the theoretical and methodological orientations that guided the development of this study protocol to the introduction (page 5). (Please note that all page numbers refer to the “tracked changes” version of the revised manuscript.)

Given that many of the field sites and samples included in this study have undergone almost no systematic, empirical study of conceptual development generally, let alone with a focus on religious concepts, we employed these methods as a starting point for what we hope to be a long-term, multi-method collaboration as a network.

- The study repeatedly claims it wants to study children's lived experiences, yet it seems to be more about their theological opinions than lived experiences. I believe that children's religious thinking and behaviour are even more determined by situational context than adults' thinking and behavior. I find the tasks appropriately accommodated to children in the sense of their operation, yet I feel a lot of unease with the theoretical expectations, where children are just somewhat cognitively impaired adults.

First, we agree with the reviewer that this initial research project does not extensively study children's everyday experiences (even though the protocol does include questions that assess children's experiences with religious agents etc.). Rather, it aims to study children's beliefs and reasoning about the kinds of religious entities, practices, and norms they encounter in their everyday lives. As we mentioned above, we have added more about the theoretical and methodological orientations that guided the development of this study protocol to the introduction (page 5).

In designing the protocol presented here, we relied on extensive conversations with religious leaders, formal and informal educators, and other community leaders to design tasks and select stimuli that align with children's lived experiences in a given cultural-religious setting. Our goal is to embed information about situational contexts and specific religious content that is relevant to children's everyday lives so as to evoke their judgments, memories, concepts, and reasoning.

We have revised the manuscript accordingly. For instance, where the initial submission said that a primary goal was to "understand a child's lived experience" we now say that a primary goal was to "understand children's views around the kinds of religious entities, practices, and norms they encounter in their everyday lives (Rogoff et al., 2018)." In introducing research question 3, we also note how the present study can inform future studies of the specific contexts in which children learn about religion in their everyday lives: "By using open-ended questions to probe the sources of children's religious cognition and identity, these tasks can inform future studies of specific contexts in which children learn about religion, for instance by naturalistic observations of parent-child interactions, peer conversations, or school contexts."

Second, we certainly do not think of children as "cognitively impaired adults." We discuss the developmental framework and the DBN's research aims at length in a previous manuscript (Richert et al., 2022, Advances in Child Development and Behavior), which we cite in the current manuscript (pages 6), in addition to the new paragraph that briefly describes the theoretical and methodological orientations that guided the development of this study protocol (page 5).

We admit that we are not entirely sure which parts of our manuscript gave the reviewer this impression. Still, it is an impression we are eager to correct. The study includes a wide age range, both to chart the developmental progression of religious cognition across our sites and to understand how children at their developmental level grapple with religion and the supernatural. Inevitably, adults do know more than children about certain religious issues; still, we see children as active and often ingenious participants in their own religious development at every

developmental stage. In response to the reviewer's comment, we now include the following sentence before we introduce the three research questions: "The study covers a wide age range, to chart the developmental progression of religious cognition as well as to understand how children at a given developmental level grapple with the religious and the supernatural" (page 11).

- The most missing part of the study for me is the missing theorisation of cognitive-situational frames, which could be in a developmental research context probably manifested best in the problem of "play".
- I find this large and cross-culturally broad studying children's cognition oriented on religion and fiction without considering the problem of the "magic circle", i.e. the cognitive machinery responsible for maintaining situational borders between social frames of action, a greatly missed opportunity.

As we previously noted, we relied on extensive conversations with religious leaders, formal and informal educators, and other community leaders to design tasks and select stimuli that align with children's lived experiences in a given cultural-religious setting. Accordingly, we are confident that the situations presented to the children were consistent with their unique lived experience. Therefore, we anticipate that the interactions with children during this protocol reflect their experience of religion within their ordinary lives.

Indeed, we explicitly designed our protocol to allow for multiple and varied opportunities for children to reflect on their experiences, including the opportunity to justify their responses through open-ended discussions, as well as parent-child conversations to observe the transmission of information to the child. We believe that such varied interactions allow for the child to express their beliefs within the context of their lived situation. They also provide us (researchers) opportunities to assess which frame(s) children are drawing on in their reasoning about a given question or prompt.

- To sum up: I have a concern about the ecological validity of the findings, and I mostly fear two things. Firstly, the findings will be impaired by the unjustified reduction of lived human behaviour to a cognitivist paradigm of religious belief as out-of-context raptured mental representations and mechanisms. Secondly, the findings will reflect more on how adults guide and understand the children's representation of religion, than how children really navigate the landscape of the religious and non-religious things in their lives.

We thank the reviewer for engaging with this research with both generosity and a critical eye, and we truly hope that the protocol described in this manuscript will inspire other researchers to develop and apply other methods to complement the tasks we have developed in painting a nuanced picture of this complex domain of human development and experience.

We agree with the reviewer that beliefs and mental representations are only one part of children's religious and spiritual development, and adult caregivers are only one of the

influences that shape these emerging representations and identities. However, the development of beliefs and the "transmission" of beliefs from caregivers to children are the core focuses of this first wave of research from the Developing Belief Network, which is why they figure so prominently in the current protocol. As we note in our manuscript, this is because religion is a domain under which children must rely on the testimony of others, instead of learning from first-hand experience. Nevertheless, we designed the protocol to allow children to present their own views on their religious experience separate from those of their parents in the hopes of exploring potential similarities or variabilities in the information transmitted to the child, as well as the information that was encoded by the child.

Based on our extensive interviewing with the religious leaders within the community, we believe we have gone to great lengths to ensure that the stimuli and situations presented to children in the course of this study protocol are ecologically valid, tailored to the specific cultural-religious context of the sample in question. We believe that these tasks will allow us to gain insights into how children navigate the landscape of religious and non-religious things in their lives.
