# Supplementary material for: The development and diversity of religious cognition and behavior: Protocol for Wave 1 data collection with children and parents by the Developing Belief Network
Source: PLoS One. 2024 Mar 8;19(3):e0292755. doi: 10.1371/journal.pone.0292755 (PMC10923471; doi:10.1371/journal.pone.0292755)
Supplement: Attachment — Submitted filename: Response to Reviewers.pdf [file pone.0292755.s003.pdf]

Response to Reviewers

*Please note that our responses to reviewers are provided in **blue**, and all page numbers refer to the "tracked changes" version of the revised manuscript.*

Also note that we have made minor updates throughout the manuscript to reflect current network members and sampling plans as of August 2023.

Reviewer Comments

Reviewer #2:

The authors have elaborated on all the points made in the first iteration of the review process and provided clarification and amendments to the manuscript where needed and I am happy to recommend it for publication.

We sincerely thank Reviewer #2 for their interest in this research and for their constructive feedback throughout the revision process.

Reviewer #4:

Overview

The present manuscript details the protocol of a large scale, multi-national longitudinal study of children's religious cognition and behavior. It is evident that the resulting data from this protocol will provide the most comprehensive data on the development of religious cognition to date, and will provide data for future researchers to examine additional questions. I found the manuscript fascinating, and I feel the need to commend the author on the time and care they took on preparing this submission. I also should acknowledge that this is my first time reviewing a study protocol. Given that this manuscript is reporting partly on the protocol of the entire study, but partly focusing on the samples from the United States, and that data collection has started (and so modifications to the actual questions or measures is not possible), I found it challenging to determine what my comments should focus on. Therefore, please consider most of my comments suggestions.

We thank the reviewer for engaging with this research so thoroughly, and we are glad to see that the reviewer agrees that this research will yield fascinating data for current and future researchers.

Major comments

One consistent challenge that I had in making sense of this manuscript is that in my reading the manuscript is trying to do two things at once. First, it is describing the Developing Belief Network at large and describing a basic protocol and sample description for the project as a whole. Second, it is providing a more detailed protocol for specifically the sites in the United States. This means that the authors several examples of the materials and more details on the method for the sites in the United States. For me this was confusing and kept raising questions about how several issues were going to be handled for the other samples. It did not help that the authors sometimes mention how the protocol will be adapted (e.g., selection of religious entities includes adaptation for Buddhism, even though this is not one of the religious groups being recruited in the US and details on other modification), but other times do not (e.g., sample size determination). This was odd to me as several non-US sites have started or even completed data collection, meaning that many of these decisions have been made already. I assume that the authors are trying to strike a balance in this manuscript between being comprehensive but also succinct (particularly given the current length of the manuscript), however, I think a few more details in different sections would enhance the manuscript. For example, the authors mention that each site will vary across samples, which is fine, but they can add “with at least XX children per sample” or they could state in the “Sample size (US samples)” section that a similar process will be used for the other sites.

We thank the reviewer for recognizing these two goals of the manuscript and providing this feedback. We have attempted to clarify throughout the manuscript which aspects of the protocol apply to the whole network and which aspects are specific to US samples (e.g., by repeating the note that some procedures vary across samples), and we have modified our descriptions of tasks to focus on US samples. On the specific note regarding sample size, we have added the reviewer’s suggestion (“at least XX children per sample”; page 17).

Please include details on the translation process. In page 7, it says that this will be included in future manuscript. However, this is a critical component of the protocol and it is an aspect that I assume has already taken place (as 68% of the samples have started data collection). Therefore, I encourage the authors to include at least a basic description of their current approach.

The translation process has varied substantially across research teams and field sites/samples, and has been coordinated entirely by research team leaders rather than standardized across the network. We have added a brief acknowledgement of this to the manuscript (page 8).

What is the justification for the 75 participant minimum (and 100 maximum) for each sample? Why was the September 30th date selected? Is it an arbitrary date? After a certain number of months from the start of data collection? Necessary for grant reporting or for a student to graduate? Whatever the reason it is fine, but to me it seems like important information to mention in a protocol. Will similar stopping rules be used for the non-US sites? Again, I am

assuming that this decision has already been made as data collection in some sites as finished, so I am surprised this is not included in the manuscript.

Thank you for these questions; we have updated and expanded on our sampling rules for US samples in the manuscript (pages 17-18). We have also noted in the manuscript that these procedures vary across samples and are not included here; we would strongly prefer to provide sample-specific details regarding sampling, exclusion/inclusion, recruitment, compensation etc. along with additional contextualizing information for each cultural-religious group, and we do not have the space to do this in the current manuscript.

I have two questions regarding exclusions due to frequent interruptions. First, what percentage of question would cause a child's data to be excluded? How will the research team ensure consistency across the sites (the two US sites at least)? Second, what about cases in which there is interference in some questions, but there is not enough to justify the exclusion of the entirety of the child's responses. Will only the questions with interference be eliminated? Retain the response prior to the interference? These details might be worth specifying in the protocol.

We have added details about exclusion decisions for US samples (pages 20-21).

I was surprised by the lack of statistical details on the protocol. What type of modeling techniques will the researchers use? How would they determine random effects structures or handle non-convergence (if multilevel models will be used)? Will they use frequentist or Bayesian models?

After much discussion among our members, the network has agreed to preregister specific confirmatory analyses for specific empirical papers at the discretion of the lead author(s) for each empirical paper. (We have laid this out in our Authorship Guidelines, which are available as a preprint: https://osf.io/rwhjd/.) With such a wide-ranging protocol, such diverse interests represented in the network, and such a wide range of reasonable approaches to the many research questions that are relevant for each component of the protocol, we feel that it is beyond the scope of the current manuscript to provide further details about the modeling techniques for every planned analysis.

I appreciate the authors noting when certain analyses are exploratory. However, I found the sections on qualitative analyses to be lacking details. I read the authors' responses to a similar comment by another reviewer, but even if the analyses are exploratory the authors can include more details on how they plan to approach the qualitative data analysis. As it currently stands, it seems like the qualitative analyses are an after-thought, which is surprising given that there are planned analyses and hypotheses regarding the qualitative data, and that the authors present this study as a mixed-methods study. Even if there is not an existing coding scheme the authors can state the type of qualitative methods they will use. Something as broad as stating that they

will use thematic or content analysis (rather than other techniques like narrative analysis) would make the protocol more robust. If coding schemes are going to be developed, then how will this be done? Will they look at a certain percentage of the data to develop the coding scheme? Look at all the data? Will the coding scheme be developed inductively or deductively? I imagine it is a mix, as the hypotheses indicate that the authors already have some codes in mind. Will a particular code need to be present a certain number of times or mentioned by a certain percentage of participants for it to be included in the final scheme? The authors could also detail their coding and reliability process. Will there be multiple coders that independently code the specific percentage of the data to assess reliability? If so, what would this percentage be? How many coders? What reliability indicator would you use and what cut off point? How will you handle codes with low reliability, or low prevalence (which would impact reliability)? Would you use a form of consensus coding that would require no assessment of reliability? Many qualitative techniques differ in all of these factors so many of these decisions would be fine methodologically, but the authors should specify them a priori and include those details in the manuscript.

Thank you very much for raising this – qualitative analyses are, indeed, a fundamental part of this research protocol and we appreciate the opportunity to clarify this in the manuscript.

We see the process of developing approaches to qualitative analyses of these datasets as a hugely important topic of discussion, and we feel that we could not do justice to this in the current manuscript. We are committed to these analyses emerging from a long-term, iterative process of collaboration involving research team leaders, on-site staff, and additional cultural and religious experts from sites from across the network, and as of the current resubmission this process is only in the very earliest stages. Moreover, we anticipate that different standards will be applied to different parts of our dataset emerging from the various tasks that yield open-ended and otherwise "qualitative" data (regarding, e.g., the process of developing coding schemes; the process of establishing, maintaining, and assessing inter-coder reliability).

We have added a new section to the manuscript describing this perspective (pages 83-84).

Minor comments

For some tasks, such as the biological/physical/psychological properties, the authors state the length of the task as whole. For other tasks, such as the religious indicators task, the authors state the length of each block. I would recommend they are consistent on which they report, and suggest they report the length of the task as a whole.

We have revised our estimates to be more clear and consistent.

How will the authors compare the child and parent responses? Will it be done as a group? Match the children to their parent? If so, how would the authors handle siblings?

We anticipate that different sets of lead authors will take different approaches to comparing child and caregiver responses when analyzing different tasks (and even when conducting multiple analyses of the same task). For example, we are aware of subsets of network members interested in applying at least three distinct approaches to this: conducting comparisons at the group level (i.e., comparing a "child sample" with an "adult sample"), using individual caregiver data as a predictor of individual child responses, and analyzing "correspondence" between caregiver and child responses (with the degree of correspondence between responses, rather than the responses of an individual, as the outcome of interest). These different approaches will require different handling of siblings.

Regarding siblings, we also note that different samples will vary in the extent to which siblings from the same family contribute to the child sample, based in large part on demographic trends in the population of interest. Samples from the US, for example, will include some siblings; the sample from Shanghai, China, will likely include relatively few siblings; and samples from the Yucatán, Mexico, will likely consist primarily of siblings. In order to accommodate this aspect of variability across cultural-religious settings, we have chosen not to establish network-wide standards for inclusion/exclusion or network-wide analysis plans that extend to all data emerging from these datasets.

This is a minor point, but in the social essentialism task the authors include a question that involves "souls." Given the different religious backgrounds (including children that are unaffiliated to any religion) in the entire study, will all children interpret this question similarly?

We agree with the reviewer that the interpretation of the word "soul" (and its correlates across languages and religious settings) is likely to vary across samples. This is of great interest to many of the members of this network and we look forward to exploring it, e.g., through item-wise analyses of the social essentialism task and relationships between children's responses to this question, their demographic information, and other information from caregivers about their upbringing, religious experiences, etc. This item is the most "novel" and "exploratory" of the social essentialism items included in this task, and we hope that the inclusion of more "traditional," widely used indices of essentialism (i.e., items referring to inherence at birth, to blood, and to constancy over development) will help us make sense of whatever response patterns we observe.

We note that we would not have included this item if cultural experts the field sites/samples represented in the network had indicated that it would be problematic to ask in a given setting; semi-structured interviews with cultural experts and religious leaders did not raise such concerns. In the future, returning to these (or additional) cultural experts and religious leaders to aid in the interpretation of our results could be one way of understanding how "souls" were construed by participants in each sample.
